# The Development and Content Validation of the Care Partner Hospital Assessment Tool

**DOI:** 10.3390/nursrep11030049

**Published:** 2021-07-11

**Authors:** Beth Fields, Richard Schulz, Lauren Terhorst, Madeline Carbery, Juleen Rodakowski

**Affiliations:** 1 Department of Kinesiology, University of Wisconsin-Madison, Madison, WI 53706, USA; mcarbery@wisc.edu; 2 Department of Psychology, University of Pittsburgh, Pittsburgh, PA 15260, USA; schulz@pitt.edu; 3 Department of Occupational Therapy, University of Pittsburgh, Pittsburgh, PA 15260, USA; lat15@pitt.edu (L.T.); jur17@pitt.edu (J.R.)

**Keywords:** hospital, screening, caregiving, aging, instrumentation

## Abstract

Background/Objectives: When aging adults are hospitalized due to a major health event, they often turn to care partners (‘family members or friends’) for support. Assessment of care partners’ needs during hospital care may be important to inform and target information and skills training that will equip them to fulfill caregiving tasks for the aging adults. The objectives of this study were to develop and complete content validation of the Care Partner Hospital Assessment Tool (CHAT). Methods: Based on standard instrumentation methodology and an assessment framework recommended by the National Center on Caregiving at the Family Caregiving Alliance, three steps were followed to develop and validate CHAT: (1) generation of a 24-item tool grouped into three content domains (background, plans and preferences, skills and supports), and a survey by a multidisciplinary team, (2) administration of an online survey of care partners and experts, and (3) assessment of item and scale-content validity indices (I-CVI and S-CVI). Results: A total of four care partners that provide unpaid care to a family member or friend age 65 years or older with a chronic illness or disability either before or after a hospitalization, and 19 leading experts in gerontology, caregiving, and health services completed an online survey in English. Twenty-two items were accepted by having an I-CVI at or above the acceptable 78% cut point; the S-CVI for the tool was 85%. Most revisions to the tool were associated with modifying or clarifying language within each item. For example, participants shared the following open-ended suggestions for revising CHAT: (1) change the “do you prefer” sentence stem to “do you want” (*n* = 12), define “training” (*n* = 6), and (2) allow care partners to provide an unsure response (*n* = 5). Conclusion: CHAT may be a promising way to increase health care practitioner’s understanding of care partners’ backgrounds, preferences and plans, and potential information or training needs during a patient’s hospital stay. Initial evaluation of CHAT reveals strong conceptual development and content validity.

## 1. Introduction

Many aging adults experience a major health event that requires medical, surgical, or rehabilitative hospital services. The health and functional status of these aging adults are often exacerbated by early hospital discharge to home due to advances in medical technology and care management practices [1]. As a result, aging adults often turn to care partners (‘family members and friends’) for support during and after their hospitalization. Consensus recommendations and guidelines have long recognized the importance of including care partners in the hospitalization process, and have further called for care partner assessment to determine what types of information and skills training they might need to successfully complete their caregiving responsibilities [2,3].

We conducted a systematic review to identify and characterize existing assessments for care partners of aging adults [4]. We found more than 66 unique care partner assessments, yet only 7% (*n* = 4) were designed for the hospital setting. Care partner assessments were condition-specific (i.e., cancer), and did not capture the multiple needs of care partners or identify the information and skills training they might need. For example, the Family Inventory of Needs [5] was designed to measure the care needs of families supporting patients with advanced cancer, and the McMaster Family Assessment Device [6] has been used to assess the preferences and values of care partners involved in their loved one’s hospital care. Further, the psychometric evaluation of these assessments was limited or not reported. Of the four assessments designed for the hospital setting, only two reported good reliability and validity, and none reported on the clinical utility of the assessment. These findings demonstrated a need for a valid and reliable multidimensional assessment that identifies care partner information and skills training needs that should be prioritized before the aging adult is discharged. The downstream effects of a trained care partner before the aging adult is discharged has the potential to influence outcomes for both the care partner and the aging adult. An efficient approach to achieving this goal is to administer a brief screening tool, as early as possible during the aging adult’s hospital stay, to identify care partners who should receive further evaluation and intervention [7].

The objectives of the current study were to develop and establish content validity for the Care Partner Hospital Assessment Tool (CHAT). As a result of this work, health care systems will have a screening tool that identifies the needs of care partners and guides practitioner’s clinical decision-making for information and skills training during hospital care. Most importantly, care partners of hospitalized aging adults can expect to receive recognition and tailored information and skills training with the help of CHAT.

## 2. Methods

Our study consisted of three steps to develop and establish content validity for CHAT: (1) item generation and survey development, (2) participant recruitment and survey administration, and (3) content validity assessment. These steps follow a standard methodology to develop and establish content validity in a new assessment [8]. The Institutional Review Board at the University of Wisconsin-Madison approved the study protocol.

### 2.1. Step 1: Item Generation and Survey Development

Initial items for CHAT were generated by our study team with several rounds of development and consensus-building. Our study team, all with content and methodology expertise, included two occupational therapists and geriatric health services researchers (JR, BF), one social psychologist and caregiving researcher (RS), and one biostatistician and psychometrician (LT). We reviewed findings from our systematic review and the assessment framework recommended by the National Center on Caregiving at the Family Caregiving Alliance to inform the development of our initial items [4,9]. We selected the assessment framework to help guide the development of our initial CHAT items because it emphasizes that quality care results from identifying and assessing multiple needs of care partners. Representative domains from the framework included the following: context; care partner’s perception of health and functional status of the aging adult; care partner values and preferences; well-being of the care partner; skills, abilities, and knowledge to provide care to the aging adult; and potential resources that care partner could choose to use. Context includes information related to the physical environment, financial status, caregiving relationship, and duration of caregiving. Care partner’s perception of health and functional status of the aging adult captures information on activities of daily living, cognitive and/or behavioral impairments, medical procedures, and instrumental activities of daily living. Care partner values and preferences includes information such as their willingness to assume and accept care responsibilities and disposition for scheduling and delivering care. Well-being of the care partner refers to their self-rated health conditions and symptoms. Skills, abilities, and knowledge to provide care to the aging adult includes information related to the care partner’s confidence and competence to deliver care. Potential resources that the care partner could choose to use encompasses information on formal and informal social support services, coping strategies, and financial resources. Together, these domains represent the complexities, dynamic nature, and influence of the broad range of assistance care partners provide to aging adults that have unique characteristics, conditions, and comorbidities. To request a copy of the initial CHAT, email the corresponding author.

Our study team created a survey using Qualtrics to evaluate content validity. The survey consisted of an initial item pool representing the assessment framework domains, and questions that asked participants to (1) rate the clarity and relevance of each item on a 5-point Likert scale (1 = not at all relevant to 5 = extremely relevant), (2) provide open-ended feedback regarding content and wording immediately after each rating question, and (3) share suggestions for additional content that was overlooked in the initial item pool. Rating responses were dichotomized as ‘agree’ for those who reported 3 (somewhat relevant) or greater for each item. This cut-point follows the typical recommendation for computing content validity [10].

### 2.2. Step 2: Participant Recruitment and Survey Administration

Purposive sampling was used to recruit at least 10 participants comprising care partners and leading experts in gerontology, caregiving, and health services. This sample size exceeds the general suggestion of five experts for content validation [8]. To be eligible for step 2, care partners had to (1) provide unpaid care to a family member or friend age 65 years or older with a chronic illness or disability either before or after a hospitalization, (2) be at least 18 years or older, and (3) speak or understand written English. Experts had to (1) have at least five years of professional experience in gerontology, caregiving, or health services, and (2) speak or understand English. No participants were excluded based on sex, race, or ethnicity. Completing the Qualtrics survey served as the participant’s consent to participate in the study.

Once the survey was approved by the study team, participants were contacted via email and invited to participate electronically. The survey remained open for six weeks, and our study team contacted participants up to three times to encourage them to participate. No incentives were provided to participants for completing the survey.

### 2.3. Step 3: Content Validity Assessment

Descriptive statistics (e.g., means, standard deviations, percentages) were used to characterize the sample. Item-content validity index (I-CVI) and scale-content validity index (S-CVI) were calculated for the item pool. I-CVI was calculated by taking the number of participants in agreement based on the dichotomized response scale and dividing it by the total number of participant responses. S-CVI was calculated by taking the average of the I-CVI and dividing it by the total number of items. Acceptable agreement for I-CVI was set at 0.78 or higher, and for S-CVI at 0.80 or higher [10]. The study team used the I-CVI and any open-ended responses from the survey to guide them in revising, deleting, or substituting CHAT items.

## 3. Results

### 3.1. Item Generation

We identified three relevant content domains comprising twenty-four items (the number of items in each domain is shown in parentheses): background (6), preferences and plans (8), and skills and supports (10) based on the literature review and framework recommendations. In the background domain, care partners provide contact information and basic demographics, including age, sex/gender, relationship to the patient, living with patient, and duration of caregiving. In addition to sharing background information, care partners answer questions about their anticipated plans and preferences while caring for someone during and after hospitalization. In the skills and supports domain, care partners identify what information and training needs they may have to fulfill caregiving responsibilities after someone is discharged from the hospital. Care partners select “Yes” or “No” for all items in the preferences and plans, and skills and support domains.

### 3.2. Survey Participation

Of the 72 identified care partners and leading experts in gerontology, caregiving, and health services, 23 completed the survey (Table 1). Thus, the overall response rate for the survey was 32%.

### 3.3. Validation of CHAT

We found good agreement between participants; the S-CVI for CHAT was 85%. I-CVI for the CHAT items from the preferences and plans and skills and supports domains ranged from 65.2 to 95.6, with five falling below the acceptable 78% cut point (Table 2). More than 70% of participants thought CHAT would help identify care partners’ needs during hospital care. In addition, 74% of participants reported that CHAT was the right length for the hospital setting.

At the end of the survey, participants were invited to share general open-ended comments on CHAT. The most prominently identified suggestion was that we modify the “do you prefer” sentence stem to be more direct by using “do you want” (*n* = 12), followed by replacing “training” with “information or training” (*n* = 6), allowing care partners to “provide an unsure response in case they do not know what their caregiving responsibilities may be at the time of completion” (*n* = 5), simplifying item on gender in background domain by “reducing the number of response options” (*n* = 3), and adding more instructions to the start of CHAT to “clarify the meaning of yes or no response options” (*n* = 2).

## 4. Discussion

In light of the increasing aging population’s reliance on care partners for assistance both during and after hospitalization, the purpose of our study was to create and refine a screening tool that objectively records the needs of care partners, and in turn, guides information and training provided by skilled practitioners during hospital care. The resultant 22-item tool improves on existing approaches to care partner assessment in two important ways. First, we developed CHAT to be a multidimensional screening tool. Second, the tool takes into account real-world clinical considerations unique to the hospital setting and has strong evidence of content validity.

While many care partner assessment tools exist, most have been designed for specific population groups (e.g., cancer and stroke) and tend to focus on one caregiving domain (e.g., consequences of caregiving) [4]. Given the diverse experiences and situations of care partners of aging adults [2], our study team referred to the recommended assessment framework from the National Center on Caregiving at the Family Caregiving Alliance [9] to help ensure that CHAT is generalizable and able to capture multiple caregiving needs. Previous studies have found that health care partitioners rarely ask whether care partners are willing and able to fulfill caregiving responsibilities [11,12]. CHAT includes both a ‘preferences and plans’ and a ‘skills and supports’ domain to ensure care partner willingness and ability. For example, in the preferences and plans domain, we revised language to offer care partners a choice in taking on caregiving responsibilities both during and after the patient’s hospitalization. This change will assist practitioners in identifying the appropriate care partner to include in the patient’s care planning [13]. Given that so few care partners report receiving information or training related to their caregiving role [14], most participants recognized the value of CHAT as a way of determining if further assessment and information and training is necessary. However, a primary concern of several participants was the amount and clarity of the instructions for the response options in the skills and supports domain. To address these concerns, the revised CHAT includes more instructions on when a care partner should select either the yes, no, or unsure response options. For example, care partners are instructed to select no if they do not need information or training, or if the particular topic is not relevant to their caregiving situation.

An important result from our study is the excellent content validity of CHAT, which demonstrates that the items adequately capture the potential needs of care partners of hospitalized aging adults. Excellent content validity is attributed to “strong conceptualizations of constructs, good items, judiciously selected experts, and clear instructions regarding the underlying constructs and rating tasks” [10]. The majority of care partners and leading experts reported that CHAT would help identify care partners’ needs and be brief enough to be utilized in hospital settings. The revised tool is ready for feasibility and acceptability testing by health care teams familiar with geriatric services and discharge planning processes. To increase adoption and integration of CHAT into usual hospital care workflow, implementation frameworks such as the Consolidated Framework for Implementation Research (CFIR) [15] should be used to identify barriers and guide refinements of the tool and its administration.

Limitations of note include the web-based survey and lack of diversity among participants [16]. To address these limitations, future research should (1) use in-person interviews with health care practitioners who would administer and interpret the tool as well as care partners who would respond to the tool, and (2) assess the tool’s applicability for diverse populations. Examination of psychometric properties of CHAT, such as construct validity and test–retest reliability is also warranted.

In conclusion, CHAT is a promising tool for increasing health care practitioner’s understanding of care partners’ backgrounds, preferences and plans, and potential information or training needs during a patient’s hospital stay. Care partners that are well-prepared have potential to increase not only their health and well-being, but also help improve outcomes for aging adults during and after hospitalization.

## Figures and Tables

**Table 1:** Demographic characteristics of participants.

Characteristic	*n* (%)
Care partner sex (female)	3 (75%)
Expert sex (female)	12 (63%)
Expert affiliation	
Academic	6 (32%)
Academic Medical	9 (47%)
Industry	2 (11%)
Government	2 (11%)
Location in USA	
Northeast	14 (61%)
Southwest	1 (4%)
West	1 (4%)
Southeast	3 (13%)
Midwest	4 (17%)

**Table 2:** I-CVI scores, open-ended responses from participants, and study team revisions for CHAT.

CHAT Item	Number of Participants in Agreement	I-CVI	Open-Ended Responses	Study Team Revisions to Open-Ended Response
*Preferences and Plans Domain*				
Do you prefer to be the health care team’s contact person on behalf of the patient?	22	96%	“I think if they are not the contact person, the patient, not the contact person, should identify the next person.”	We include instructions to indicate that the patient should identify any and all care partners before administering CHAT.
2. Do you prefer to provide support to the patient during hospital care?	14 ^a^	67%	“The term ‘support’ might need slight clarification or expansion. What kind of ‘support’?”	We provide examples of support, including physical, social/emotional, and health care decisions and advocacy.
3. Do you prefer to provide support to the patient after discharge from hospital?	19 ^b^	86%	See open-ended response from item 2.	See study team revision from item 2.
4. Do you prefer to learn from health care team about patient’s condition?	18	78%	“I might be confused if I were the care partner as to whom else, I would learn from if not the health care team.”	We ask care partners if they want to learn from the health care team about the patient’s condition.
5. Do you prefer to be present when care is provided to the patient?	16	70%	N/R	N/A
6. Do you prefer to have access to the patient’s electronic medical records?	16	70%	“How will issues of HIPAA and privacy and permissions be worked out for the care partner to have this level of access and to speak for patient?”	We specify that consent from the patient is needed for care partners to have access to their electronic medical records.
7. Do you prefer to participate in decision-making about the patient’s care?	22	96%	N/R	N/A
8. Do you prefer to participate in the medical and nursing treatments for the patient?	15	65%	“There are both situations in which participating in medical and nursing care would be optimal but doesn’t occur, and situations in which the care partner would prefer not to participate but doesn’t actually have a choice.”	We removed this item because item 18 captures pertinent information about medical and nursing task needs of care partner.
***Skills and Supports Domain***				
9. Do you need training focused on understanding the patient’s health condition(s)?	19 ^b^	86%	N/R	N/A
10. Do you need training focused on managing patient’s medication(s)?	22 ^b^	100%	N/R	N/A
11. Do you need training focused on discussing issues with the patients’ health care team?	21 ^b^	95%	“Define issues. What kind of issues?”	We removed this item and modified item #7 to denote ‘discuss health care decisions about the patient’s care’.
12. Do you need training focused on helping the patient with personal care, such as dressing, bathing, or feeding?	21 ^b^	95%	“You ask about basic ADLs and IADLs. No where do you ask if they need training on assisting with mobility and transfers.”	We include ‘mobility’ as an example of personal care.
13. Do you need training focused on fulfilling household tasks for the patient, such as shopping, managing personal finances, arranging for outside services, or providing transportation?	16 ^b^	73%	“Make clear that the check boxes refer to support/training needed by the care partner.”	We list IADLs based on increasing level of complexity (shopping, transportation, arranging for medical appointments, managing personal finances). We removed ‘arranging for outside services’ because item # 16 contains this information.
14. Do you need training focused on using assistive devices, such as trach, G-tube, pumps, oxygen, wheelchair, walker, or lift, with the patient?	20 ^b^	91%	“Add more options or provide examples of what type of pumps we are referring to. In addition, should we also put (feeding tube) next to G-tube.”	We include ‘feeding tube’ as an example of a G-tube.
15. Do you need training focused on preparing the patient’s home prior to discharge, such as installing grab bars, moving furniture, or purchasing adaptive equipment?	20 ^b^	91%	“The questions tend to presume that a care partner is able to do at least several of these activities and even if they would like training that does not mean they ‘should’ be doing it.”	We provide broader language to represent care partners that could, as well as those that should not, prepare the patient’s home prior to discharge.
16. Do you need training focused on locating community-based services, such as support groups and recreational activities in your local area?	19 ^b^	86%	N/R	N/A
17. Do you need training focused on discussing advance care directives with the patient, which are legal documents that allow a person to indicate their preferences for medical care should they be unable to make decisions for themselves (e.g., coma, permanently unconscious, end-of-life)?	21 ^b^	95%	N/R	N/A
18. Do you need training focused on performing medical and nursing tasks for the patient, such as wound care or giving injections?	19 ^b^	86%	“My only suggestions would be to move this item up in the list so that it falls more in the category of medical care and less within the home and community care domain.”	We rearranged the order of items presented in CHAT.

Note: N/R = no response; N/A = not applicable; ^a^ = 21 participants responded; ^b^ = 22 participants responded.

## Data Availability

The data presented in this study are available on request from the corresponding author.
